# Lung ultrasound aeration assessment: comparison of two techniques

**DOI:** 10.1186/cc14302

**Published:** 2015-03-16

**Authors:** S Mongodi, F Mojoli, A Stella, I Godi, G Via, G Tavazzi, A Orlando, B Bouhemad

**Affiliations:** 1 Fondazione IRCCS Policlinico S. Matteo Hospital - University of Pavia, Italy; 2 Centre Hospitalier Universitaire Dijon, France

## Introduction

Lung ultrasound (LUS) allows semiquantification of lung aeration in PEEP trials [1], pneumonia [2] and weaning [3]. LUS score is based on number/coalescence of vertical artifacts (B-lines) in longitudinal scan (LONG) [4]: the pleura is identified between two ribs and its visualization limited by intercostal space (ICS) width. We hypothesized that a transversal scan (TRANSV) aligned with ICS would visualize longer pleura and a higher number of artifacts, with better assessment of loss of aeration (LoA).

## Methods

LONG and TRANSV were performed in six areas per lung (anterior, lateral and posterior, each divided into superior and inferior). Once LONG was performed, TRANSV was obtained by a probe rotation until the ribs disappeared. We considered pleural length, B-line number/coalescence, and subpleural/lobar consolidations. LUS score was assigned: 0 normal lung, 1 moderate LoA (≥3 well-spaced B-lines), 2 severe LoA (coalescent B-lines), 3 complete LoA (tissue-like pattern).

## Results

**Figure 1:**
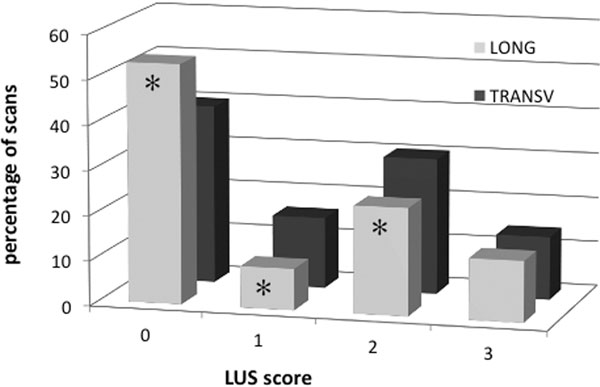
**LUS scores**. *P <0.01 TRANSV versus LONG.

We enrolled 38 patients (21 males, age 60 ± 16 years, BMI 24.7 ± 4.7 kg/m^2^) corresponding to 456 ICSs. In 63 ICSs, a tissue-like pattern was visualized in both techniques. In the other 393, LONG versus TRANSV pleural length was 2.0 ± 0.6 cm (range 0.8 to 3.8; variance 0.31) versus 3.9 ± 0.1 cm (range 3.0 to 4.3; variance 0.1) (*P *< 0.0001), B-lines per scan were 1.1 ± 1.6 versus 1.8 ± 2.5 (*P *< 0.0001), coalescent B-lines were detected in 24 versus 30% (*P *< 0.05) and subpleural consolidations in 16 versus 22% (*P *< 0.05), respectively. LUS scores' prevalence significantly differed in LONG versus TRANSV (Figure 1).

## Conclusion

TRANSV visualizes significantly longer pleura and greater number of artifacts useful for lung disease assessment.
